# The first *Helicobacter pylori*‐induced Guillain–Barré syndrome in Sudan

**DOI:** 10.1002/ccr3.8204

**Published:** 2023-11-14

**Authors:** Ayman Ahmed, Sarah Misbah EL‐Sadig, Hala Fathi Eltigani, Felix Bongomin, Emmanuel Edwar Siddig

**Affiliations:** ^1^ Swiss Tropical and Public Health Institute (Swiss TPH) Allschwil Switzerland; ^2^ University of Basel Basel Switzerland; ^3^ Institute of Endemic Disease University of Khartoum Khartoum Sudan; ^4^ Faculty of Medicine University of Khartoum Khartoum Sudan; ^5^ Sirius Training and Research Centre Khartoum Sudan; ^6^ Department of Medical Microbiology and Immunology, Faculty of Medicine Gulu University Gulu Uganda; ^7^ Faculty of Medical Laboratory Sciences University of Khartoum Khartoum Sudan; ^8^ Department of Medical Microbiology and Infectious Diseases, ErasmusMC University Medical Center Rotterdam Rotterdam The Netherlands

**Keywords:** autoimmune and demyelinating neuropathy, critical care medicine, emergency medicine, Guillain–Barré syndrome, *H*. *pylori*, infectious disease, neurology

## Abstract

**Key Clinical Message:**

This case report highlights the role of *Helicobacter pylori* infection in the development of GBS. Healthcare providers should consider *H*. *pylori* in their differential diagnosis for patients with neurological syndromes.

**Astract:**

We report the first known case of Guillain–Barré syndrome (GBS) associated with *Helicobacter pylori* infection in Sudan. This case highlights the role of *H*. *pylori* infection in the development of GBS. It also emphasizes the importance of wide screening of different endemic infections for patients with neurological syndromes for early detection and improves the case management in resource‐limited settings like Sudan. Further research is needed to better understand the underlying mechanisms of *H*. *pylori*‐inducing neurological disorders.

## Graphical abstract

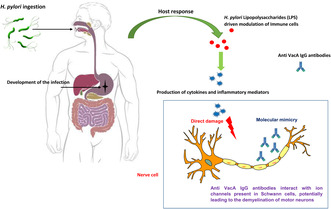

## 1 INTRODUCTION

Guillain–Barré syndrome (GBS) is a health condition that is characterized by acute inflammation that affects the peripheral nervous system.^1^ GBS is an autoimmune, neuro‐inflammatory disease that occurs when the immune system mistakenly targets the peripheral nerves due to the resemblance between certain epitopes of infectious agents and the body's own nerve tissues, in a process called molecular mimicry.^2^ The infectious agents that can trigger the development of GBS are diverse, including viruses such as severe acute respiratory syndrome coronavirus 2 (SARS‐CoV‐2), Hepatitis E and B, Influenza A, as well as arboviruses like dengue, West Nile, and Zika viruses. It also includes bacterial infectious agents such as *Campylobacter jejuni* and *Helicobacter pylori* (*H*. *pylori*), as well as parasitic infections like malaria and Leishmania, which can be involved in the development of GBS.^1^, ^2^, ^3^, ^4^

The immune response that is triggered by these infections can lead to the characteristic demyelination and dysfunction of both motor and sensory nerve fibers, which is observed in GBS.^5^ Interestingly, around two‐thirds of GBS patients may experience respiratory or gastrointestinal symptoms before the onset of the typical manifestation of GBS condition.^5^ Surprisingly, a third of patients may not exhibit any symptoms until GBS presents clinically.^5^ This highlights the challenges faced in the diagnosis, surveillance, and early detection of GBS. Consequently, the global, regional, and national burden of GBS is underestimated, particularly in resource‐limited settings like Sudan.

*Helicobacter pylori* is a widespread gram‐negative bacterium with a distinctive spiral shape that is responsible for causing serious chronic bacterial infections worldwide.^6^ *Helicobacter pylori* infection has been identified as a major contributor to various health conditions such as chronic gastritis, peptic ulcers, gastric mucosa‐associated lymphoid tissue (MALT) lymphoma, and gastric adenocarcinoma. Unfortunately, in 2020 alone, there were over 1 million new cases of gastric cancer and nearly 800,000 deaths attributed to *H*. *pylori*‐related diseases, making it the third leading cause of global cancer deaths.^7^ In addition to its impact on the stomach, *H*. *pylori* infection can also affect other areas of the body. While the exact mechanisms are still being studied, there have been reports linking *H*. *pylori* infection to various neurological disorders, including Parkinson's disease, GBS, Alzheimer's disease, and stroke.^8^, ^9^

In Sudan, the prevalence of *H*. *pylori* is rapidly growing during the recent years; this rapid growth is mainly influenced by climate change that increases the suitability of environment for the spread and establishment in new areas.^10^, ^11^ In this communication, we report the first case of GBS associated with *H*. *pylori* infection from Sudan.

## 2 CASE PRESENTATION

An adolescent male, 19 years old, presented with a sudden onset of sensory loss and muscle weakness specifically in his right lower limb for 2 weeks. Also, before the occurrence of the sensory loss and muscle weakness, the patient reported a persistent burning pain in his stomach, intermittent abdominal pain, and vomiting that had been occurring for a couple of weeks. No known history of a similar condition or pre‐existing chronic illnesses, such as diabetes, peptic ulcer, or gastroesophageal reflux disease (GERD), were reported. Additionally, there were no notable findings in the family's medical history. Furthermore, it is important to note that the patient has reported no previous record of medications.

He had a pulse rate off (89/min), respiratory rate (19/min), blood pressure (91/62), and temperature (37°C). Systemic examinations, including cardiovascular (CVS), respiratory, and endocrine, were all within the normal range. Upon examination, the muscle power in his right lower limb was rated at 1/5, while the strength in his other limbs remained unaffected at 5/5. A noticeable decrease in deep tendon reflexes was also observed in the affected lower limb compared with the unaffected side.

As a result of these symptoms, the patient was admitted to Omdurman teaching hospital, Khartoum, Sudan. Brain computed tomography (CT) scans and spinal cord magnetic resonance imaging (MRI) were performed, but no abnormalities were found. No neurological disorders such as cognition dysfunctions or conscience impartment were detected or reported.

Laboratory examinations were conducted, including a complete blood cell count with differential counting. The results showed a white blood cell count of 11,300/μL, hemoglobin of 12.4 g/dL, platelet count of 315,000/μL, neutrophils at 53.3%, lymphocytes at 38.9%, monocytes at 4.6%, eosinophils at 2.8%, and basophils at 0.4%. Alanine aminotransferase and serum creatinine levels were also assessed and found to be 80 IU/L and 0.57 mg/dL, respectively. Viral screening by Enzyme immunosorbent assay (ELISA), for locally endemic viruses of public health concerns, including human immunodeficiency virus (HIV); Hepatitis A, B, C, D, and E, Cytomegalovirus (CMV); Epstein–Barr virus (EBV); endemic arboviruses of public health importance such Crimean‐Congo Hemorrhagic fever (CCHF), dengue, Zika, and Rift Valley fever (RVF) virus all were negative. Additionally, *Campylobacter jejuni* immunochromatography (ICT) and malaria were negative. Furthermore, we performed stool antigen rapid test and urea breath test and both were positive for *H*. *pylori* infection.

Cerebrospinal fluid (CSF) examination revealed 0/μL monocytes, while the random blood glucose level was measured at 4.6 mmoL/L, and the protein level was found to be 275.3 mg/dL, indicating albuminocytologic dissociation. CSF gram stain was done and the result was negative. Investigations of nerve conduction have demonstrated evidence of demyelinating neuropathy with dysfunction of motor and sensory nerve fibers, confirming the involvement of GBS that is associated with *H*. *pylori* infection.

The patient was then treated with intravenous immunoglobulin at a dose of 0.4 mg/kg per day for 5 days. Additionally, a regimen consisting of omeprazole 20 mg BID, clarithromycin 500 mg BID, and amoxicillin 1000 mg BID was administered for 14 days to treat the *H*. *pylori* infection. Over the following 2 weeks, the patient experienced a gradual improvement in his clinical condition and muscle strength. A second CSF examination was performed 2 weeks after admission and revealed 11/μL monocytes and a protein level of 83.2 mg/dL, indicating positive progress in the treatment.

## 3 DISCUSSION

Here, we report the first case of *H*. *pylori*‐induced GBS in Sudan. This is one of the very first evidence from the field about the involvement of *H*. *pylori* in the development of GBS. Despite the globally increasing prevalence of *H*. *pylori*, the diagnostic capacity in the country remains underdeveloped. Therefore, the diagnosis of such infections with various clinical manifestation is challenging for healthcare providers with limited experience and resources. In the current case, the diagnosis was particularly challenging mainly for three reasons; first, the diagnostic capacity in the poor country is very limited with lack of in‐country sequencing services and lack of integrating molecular tools in most of healthcare facilities throughout the country.^12^

In countries like Sudan, where there is a heavy and rapidly increasing burden of various infectious diseases that are involved in the development of GBS, identifying GBS and detecting the underlying causes are challenging for healthcare providers with the current diagnostic capacity.^1^, ^13^, ^14^ Most of these diseases have similar symptoms, and their burden and spread in the country is rapidly growing due to climate change, conflicts, and increased size of population lives in humanitarian crisis.^15^, ^16^, ^17^, ^18^, ^19^ Major GBS‐associated infections that are prevalent in Sudan include COVID‐19,^20^ Hepatitis E,^14^, ^21^ Epstein–Barr virus (EBV),^22^, ^23^ malaria,^24^ leishmaniasis,^25^ *Mycoplasma* spp.,^26^ and several endemic arboviruses.^12^ Arboviral diseases that are associated with GBS and endemic in Sudan include Chikungunya, CCHF, dengue, yellow fever, and RVF viruses.^27^, ^28^, ^29^, ^30^, ^31^, ^32^ Therefore, we have tested the patient for these infections and other infections that associated with GBS development such as HIV, hepatitis A, B, C, D, and E, cytomegalovirus (CMV), and *Campylobacter jejuni*, and they were all negative.^33^, ^34^, ^35^, ^36^ In such areas, where multiple infectious diseases are prevalent, it is important to conduct thorough investigations of infections to accurately identify the causative agent and improve the case management of GBS and treating the underlining condition. GBS is the most common cause of acute flaccid paralysis; however, it is challenging to diagnose in limited capacity and resources settings, as it requires a complete medical history, neurological examination, electrophysiological test, and cerebrospinal fluid analysis.^1^, ^2^ Therefore, to better understand and control GBS in the country, it is necessary to invest in building the diagnostic capacity and improving the training of healthcare providers.

*Helicobacter pylori* is a gram‐negative bacterium known for its role in triggering autoimmune gastrointestinal disorders.^37^ However, recent research has also implicated *H*. *pylori* in autoimmune sequelae and peripheral neuropathies, suggesting its potential involvement in GBS.^38^ GBS is a severe autoimmune demyelinating disorder of peripheral nerves, often triggered by postinfection events. While *C*. *jejuni* is commonly associated with GBS, however, laboratory tests excluded *C*. *jejuni* infection in this case; this supports the growing evidence about the involvement of *H*. *pylori* infection in the development of GBS.^39^ Neurological complications of *H*. *pylori* infections reported so far include stroke, Parkinson's disease, Alzheimer's disease, and the GBS.^8^, ^9^

**FIGURE 1:**
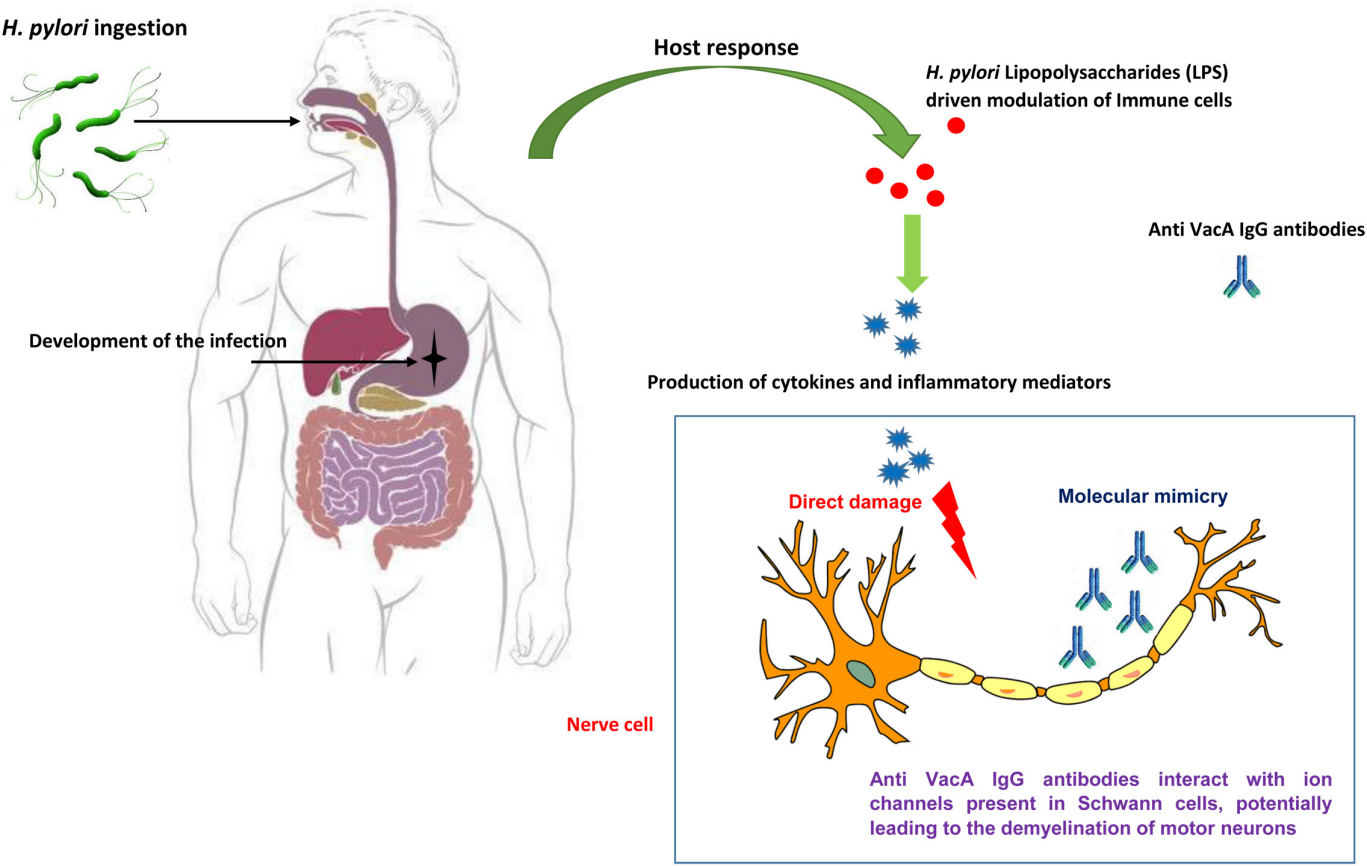
Illustrates the likely pathways through which *Helicobacter pylori* may trigger the development of Guillain–Barré syndrome.

Several hypotheses were developed about mechanisms of *H*. *pylori*‐inducing GBS; however, up to date, the exact mechanism is still unclear. A potential mechanism for the pathogenic connection has been suggested^39^; the concept of molecular mimicry between *H*. *pylori* lipopolysaccharides (LPS) and peripheral nerve gangliosides.^40^ Furthermore, research revealed elevated levels of IgG antibodies against *H*. *pylori* Vacuolating cytotoxin A (VacA) in the cerebrospinal fluid of GBS patients.^41^ Interestingly, a sequence similarity between VacA and the human ATPase A subunit was revealed recently.^41^ This finding suggests that antibodies targeting VacA could potentially interact with ion channels present in Schwann cells, potentially leading to the demyelination of motor neurons. Further investigation is required to fully characterize the role of these antibodies in the development of motor neuron demyelination. In Figure 1, we are explaining the potential mechanism of *H*. *pylori*‐inducing GBS (Figure 1).

Our case report provides essential evidence from the field about the role of *H*. *pylori* in the development of GBS and urges further investigations to estimate the actual burden of *H*. *pylori*, the associated GBS, and the mechanism of *H*. *pylori*‐induced GBS. There is an extremely urgent need for improving the diagnostic capacity in countries like Sudan, where heavy burden of wide range of infectious diseases that are involved in the development of GBS.^1^, ^13^, ^14^ Particularly, due to this similarity it is very common that infections are initially misdiagnosed and treated for the wrong infection.^42^, ^43^, ^44^, ^45^ Therefore, it might be useful to explore the feasibility of leveraging the use of Artificial Intelligence (AI) in the diagnosis and surveillance of infectious diseases, particularly in such complicated settings.^46^ Moreover, research is needed to investigate and develop effective prevention and control measures to reduce the spread and burden of *H*. *pylori* and the associated manifestations. However, to reduce the burden of GBS, countries must invest in implementing effective prevention and control strategies for the endemic infectious diseases that are involved in the development of GBS. These strategies include effective vector surveillance and control, vaccinations, and improved diagnostic and surveillance systems.^42^, ^43^, ^44^, ^45^, ^46^, ^47^, ^48^, ^49^, ^50^, ^51^

In conclusion, this case report indicates the role of *H*. *pylori* infection in the development of GBS. While GBS is commonly triggered by different infections, still there is a major gap in knowledge about the role of *H*. *pylori* in its development. Further research is needed to fully understand the exact mechanisms by which *H*. *pylori* infection may contribute to the development of GBS. This will help improving the prevention and control measures as well as the case management. Additionally, given the growing burden of *H*. *pylori*‐related diseases and health conditions, including gastric cancer, it is crucial for healthcare providers to consider the potential neurological complications associated with this infection during their differential diagnosis process.

In resource‐limited settings like Sudan, where multiple infectious diseases with similar clinical manifestations prevail, it is essential to implement a transdisciplinary One Health strategy to improve human, animal, and environment health. By improving our understanding of the burden and prevalence of GBS and the involved infections including *H*. *pylori*, we can develop cost‐effective prevention and control measures for these related health conditions.

## AUTHOR CONTRIBUTIONS

**Ayman Ahmed:** Conceptualization; data curation; formal analysis; investigation; methodology; supervision; validation; visualization; writing – original draft; writing – review and editing. **Sarah Misbah El‐Sadig:** Conceptualization; data curation; investigation; methodology; supervision; validation; visualization; writing – original draft. **Hala Fathi Eltigani:** Conceptualization; data curation; formal analysis; investigation; supervision; validation; visualization; writing – original draft; writing – review and editing. **Felix Bongomin:** Methodology; validation; visualization; writing – original draft; writing – review and editing. **Emmanuel Edwar Siddig:** Conceptualization; data curation; formal analysis; investigation; methodology; resources; supervision; validation; visualization; writing – original draft; writing – review and editing.

## FUNDING INFORMATION

None.

## CONFLICT OF INTEREST STATEMENT

The author reports no conflicts of interest in this work.

## CONSENT

Written informed consent was obtained from the patient to publish this report in accordance with the journal's patient consent policy.

## Data Availability

The data that support the findings of this study are available from the corresponding author upon reasonable request.
